# The Emerging Roles of the Metabolic Regulator G6PD in Human Cancers

**DOI:** 10.3390/ijms242417238

**Published:** 2023-12-07

**Authors:** Alfar Ahamed, Rendy Hosea, Shourong Wu, Vivi Kasim

**Affiliations:** 1 Key Laboratory of Biorheological Science and Technology of Ministry of Education, College of Bioengineering, Chongqing University, Chongqing 400045, China; 2 The 111 Project Laboratory of Biomechanics and Tissue Repair, College of Bioengineering, Chongqing University, Chongqing 400044, China; 3 Chongqing Key Laboratory of Translational Research for Cancer Metastasis and Individualized Treatment, Chongqing University Cancer Hospital, Chongqing University, Chongqing 400030, China

**Keywords:** pentose phosphate pathway (PPP), glucose-6-phosphate dehydrogenase (G6PD), tumor metabolism, tumor cell proliferation, drug resistance, anti-tumor therapy

## Abstract

Metabolic reprogramming, especially reprogrammed glucose metabolism, is a well-known cancer hallmark related to various characteristics of tumor cells, including proliferation, survival, metastasis, and drug resistance. Glucose-6-phosphate dehydrogenase (G6PD) is the first and rate-limiting enzyme of the pentose phosphate pathway (PPP), a branch of glycolysis, that converts glucose-6-phosphate (G6P) into 6-phosphogluconolactone (6PGL). Furthermore, PPP produces ribose-5-phosphate (R5P), which provides sugar-phosphate backbones for nucleotide synthesis as well as nicotinamide adenine dinucleotide phosphate (NADPH), an important cellular reductant. Several studies have shown enhanced G6PD expression and PPP flux in various tumor cells, as well as their correlation with tumor progression through cancer hallmark regulation, especially reprogramming cellular metabolism, sustaining proliferative signaling, resisting cell death, and activating invasion and metastasis. Inhibiting G6PD could suppress tumor cell proliferation, promote cell death, reverse chemoresistance, and inhibit metastasis, suggesting the potential of G6PD as a target for anti-tumor therapeutic strategies. Indeed, while challenges—including side effects—still remain, small-molecule G6PD inhibitors showing potential anti-tumor effect either when used alone or in combination with other anti-tumor drugs have been developed. This review provides an overview of the structural significance of G6PD, its role in and regulation of tumor development and progression, and the strategies explored in relation to G6PD-targeted therapy.

## 1. Introduction

Cancer, a complex disease involving a multitude of cellular changes, is a major global health concern. At its core, cancer is driven by genetic mutations that lead to an uncontrolled division and growth of cells, sustained proliferation, resistance to programmed cell death, and other alterations. As highlighted in a World Health Organization report, cancer accounted for nearly 10 million deaths worldwide in 2020 [1]. Despite advancements in laboratory techniques and early screening methods, effective cancer treatments remain a major challenge. Our understanding of cancer has evolved significantly over the years, with the latest research identifying 14 hallmarks that encompass the multi-faceted nature of this disease. These hallmarks include sustaining proliferative signaling, evading growth suppressors, resisting cell death, enabling replicative immortality, inducing or accessing vasculature, activating invasion and metastasis, reprogramming cellular metabolism, avoiding immune destruction, genomic instability, tumor-promoting inflammation, unlocking phenotypic plasticity, non-mutational epigenetic engineering, inducing senescence, and polymorphic microbiomes [2]. Of these hallmarks, one of the most complex and common is the reprogramming of cellular metabolism. This process involves changes in the metabolic pathways within a cell that support rapid proliferation and resist cell death.

Metabolism, a complex network of chemical reactions that enables organisms to maintain life, is a fundamental characteristic of living organisms. It involves both catabolic and anabolic processes, where molecules are broken down to release energy and compounds are built up, requiring energy, respectively. These biochemical reactions provide the necessary building blocks for new cells and help conserve energy [3]. Glucose metabolism in modern eukaryotic cells evolved through the endosymbiosis between ancient bacteria and archaea cells, which resulted in the incorporation of mitochondria. This in turn enabled eukaryotic cells to perform aerobic respiration, a highly efficient process that, following the initial anaerobic breakdown of glucose to pyruvate, uses oxygen to break down glucose and produce adenosine triphosphate (ATP), the primary energy currency of the cell. In addition to glucose, mitochondria also play a crucial role in the oxidation and utilization of fatty acids, another important energy source [4].

Glucose metabolism involves three stages: glycolysis, the Krebs cycle, and oxidative phosphorylation. The first step, glycolysis, occurs in the cytoplasm and breaks down one glucose molecule into two pyruvates, yielding a net gain of two ATP and two nicotinamide adenine dinucleotide (NADH) molecules [5]. This anaerobic process is followed by the tricarboxylic acid (TCA) cycle in the mitochondria, which further breaks down pyruvate to produce more ATP as well as electron carriers. These carriers are used in oxidative phosphorylation, an aerobic process that generates 32 to 34 ATP [5]. In addition to these main pathways, glucose metabolism comprises the pentose phosphate pathway (PPP), which produces nicotinamide adenine dinucleotide phosphate (NADPH) and ribose 5-phosphate (R5P) for biosynthesis reactions. Collectively, these processes allow the efficient conversion of glucose into usable energy and building blocks for cells [6].

**Figure 1:**
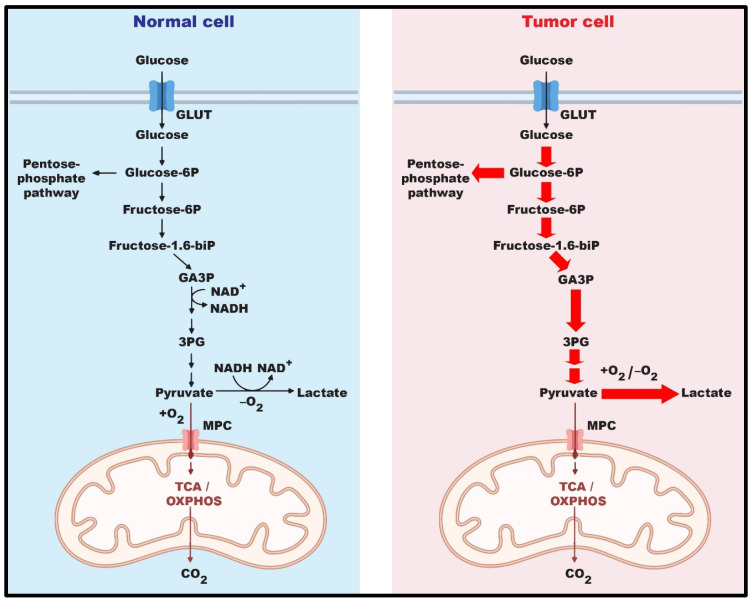
Metabolic reprogramming in tumor cells. Metabolic reprogramming is required for resolving increased demand of energy and nutrients in tumor cells for driving rapid cell proliferation. Tumor cells prefer glycolysis with enhanced lactate production rather than the typical oxidative phosphorylation regardless of the presence of oxygen.

**Figure 2:**
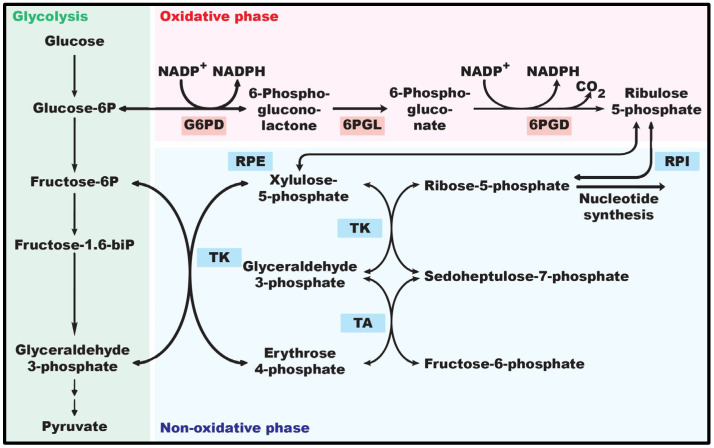
The pentose phosphate pathway. Overview of the oxidative phase PPP, the non-oxidative phase of PPP, and their link to glycolysis. RPE: ribulose-phosphate 3-epimerase; RPI: ribose-5-phosphate isomerase; TA: transaldolase; TK: transketolase.

The concept of metabolic reprogramming in tumor cells was well elucidated in The Hallmarks of Cancer in 2000 [7]. However, the unique metabolic mechanisms in tumor cells began to be deciphered much earlier, during the 1930s, through Otto Warburg’s research on the differences in glucose metabolism in normal cells and tumor cells. Warburg first observed that, even in the presence of oxygen, tumor cells preferred glucose breakdown with enhanced lactate production rather than the usual oxidative phosphorylation. This phenomenon, known as the Warburg effect, enhances the survival and proliferative potential of tumor cells by rapidly generating ATP and the glycolytic intermediates necessary for enhancing proliferation and cell growth (Figure 1) [8]. In addition to glycolysis, tumor cell metabolic reprogramming also utilizes the PPP, a shunt from the glycolysis cascade that arises from glucose-6-phosphate (G6P) and through two phases of oxidation, yielding glyceraldehyde-3-phosphate (GADP) and fructose-6-phosphate (F6P). Glucose-6-phosphate dehydrogenase (G6PD) is the rate-limiting enzyme of PPP, responsible for converting G6P into 6-phosphogluconolactone (6PGL) [9,10]. The catalytic activity of G6PD is essential in the PPP for the formation of NADPH and nucleotides via two different phases [10,11] (Figure 2). The oxidative phase releases NADPH and 6PGL in an NADP^+^-dependent way. NADPH is primarily involved in defense against reactive oxygen species (ROS) as well as lipid biosynthesis. The non-oxidative phase of the PPP yields essential molecules, including R5P, xylulose-5-phosphate, sedoheptulose-7-phosphate, erythrose-4-phosphate (E4P), glyceraldehyde-3-phosphate (G3P), and F6P. R5P provides the sugar-phosphate backbone for nucleotide synthesis [12], whereas G3P and F6P act as metabolites in the glycolysis cascade [13]. Xylulose-5-phosphate regulates the enzymes involved in the synthesis of fatty acids and triglycerides [14]. Sedoheptulose-7-phosphate functions in the generation of ribose residues for nucleotide synthesis [15]. E4P is associated with the synthesis of amino acids such as tryptophan, tyrosine, and phenylalanine [16]. The functions of PPP products are crucial for cell proliferation, cell survival, and other essential biological processes. Therefore, while glycolysis plays a significant role in providing energy, the PPP contributes to the biosynthesis of building blocks for rapidly proliferating tumor cells and to redox homeostasis [13].

G6PD plays a crucial role in maintaining redox homeostasis. It achieves this through its anti-oxidant activity, which is instrumental in balancing cellular oxidative stress [17,18]. Furthermore, G6PD is not only essential for maintaining redox homeostasis but it also plays a significant role in tumor cell metabolism. Several previous studies revealed the aberrant upregulation of G6PD in various types of cancer. This dysregulation increases the PPP flux and, subsequently, impacts tumor-related biological processes, including cell cycle regulation, DNA synthesis, DNA repair, and anti-oxidative stress response, thus generating favorable conditions for tumorigenesis, tumor progression, and drug resistance [19,20,21]. In addition, recent studies suggest that G6PD inhibitors have a significant effect on the reduction of tumor progression [22] and chemotherapy resistance [23,24]. Thus, targeting G6PD with different approaches could pave the way for novel cancer therapies. This review will elucidate and update the vital characteristics of G6PD, the redox activity of G6PD, the role of G6PD in cancer development, the regulation of G6PD in cancer, and the potential of G6PD as a therapeutic target.

## 2. Structure and Biological Role of G6PD

### 2.1. Structural Significance of G6PD

G6PD is the rate-limiting enzyme of the PPP that converts G6P into 6PGL and produces NADPH and R5P as its by-products [23]. As a housekeeping protein, G6PD is present in all types of tissues and cells, showing highly conserved homology across different species [25]. The gene that encodes for the G6PD protein is 18.5 kb long and is found in the distal arm of the X chromosome, which is proximal to the telomeric region [26]. The *G6PD* gene consists of 13 exons and 12 introns, of which the start codon ATG is found in the 2nd exon while the terminator is located in the 13th exon. The *G6PD* gene yields 1545 bp of mRNA as a transcript product, which in turn can be translated into a 59 kDa monomer protein that contains 514 amino acids [27].

The monomeric form of G6PD is catalytically inactive. The enzyme activity of human G6PD depends on the equilibrium state between the dimeric and tetrameric forms, which can be altered by environmental conditions such as pH status, as well as the levels of NADP^+^ and some metal ions [28,29]. For instance, a high-pH state favors dimer formation, whereas a higher concentration of NADPH, EDTA, and G6P favors tetramer formation [29]. Hydrophilic dimer–dimer interaction is predominantly involved in tetramer complex formation, which is the common tertiary structure of human G6PD [30]. Each dimer contains six binding sites: two substrate-binding sites for G6P, and four binding sites with one NADP^+^ each, i.e., two structural binding sites and two catalytic binding sites [27]. When G6P binds to the substrate-binding sites, it triggers a conformational change in the enzyme, which enhances the reduction of NADP^+^ in the catalytic binding site to form NADPH. The newly formed NADPH then dissociates from the catalytic binding sites. Meanwhile, unlike the NADP^+^ in the catalytic binding sites, NADP^+^ in the structural binding sites remains attached and contributes to the long-term stability of the G6PD dimer [31].

In 1956, Carson and his colleagues discovered the first human *G6PD* and determined its role in producing NADPH for ROS defense [32]. The need for structural analysis of the G6PD enzyme arose after the identification of hemolysis with G6PD deficiency in patients who had been administered certain anti-malarial drugs in the same year [33]. Thereafter, a *G6PD*-deficiency-associated cancer study was initiated by Beaconsfield in 1965 [34]. While Perisco et al. discovered the complete sequence of amino acids in 1986 by using the cloned cDNA of human *G6PD* [27], Au et al. used full-length cDNA clones with crystallographic evaluation methods and uncovered the crystallization of the G6PD tetramer in 2000 [35].

The C-terminus of the G6PD dimer has a complex interface of β-sheets and α-helix domains, which engages with the stability of the enzyme by interacting with NADP^+^. Meanwhile, the N-terminus of G6PD consists of a Rossmann-fold domain and three different conserved regions with catalytic activity [36]. The first conserved region, known as the “nine-residue peptide”, accommodates residue positions 198–206 (RIDHYLGKE) in the human G6PD enzyme that engages with G6P binding and its catalytic activity [37]. The attachment of the catalytic NADP^+^ coenzyme is linked with a conserved region called the “nucleotide-binding fingerprint”, located in residue positions 38–44 (GxxGDLA) in the human G6PD enzyme; while the third conserved region, “EKPxG”, is accommodated in residue position 170–174 between the binding sites of the G6P substrate and catalytic NADP^+^ [37]. Besides these conserved regions, other amino acid residues—especially Gly41, Asp42, Arg198, His201, Lys205, Lys171, and Pro172—also have substantial influences on the catalytic activity of human G6PD. Moreover, there are intricate associations between these amino acid residues due to a network of electrostatic interactions and hydrogen bonds, which enhance the binding affinity of a substrate or coenzyme by ensuring the appropriate orientations with their respective binding sites. Any dysregulation that occurs in these amino acid residues leads to lower binding affinity or alters the electrostatic interactions, resulting in low catalytic efficiency [36].

### 2.2. Role of G6PD in Redox Homeostasis

Previous studies revealed that cellular oxidative stress is identified as a threatening factor for aging as well as several chronic diseases, including cancer [38,39,40]. Cellular redox homeostasis is primarily regulated by reactive species (RS) [41]. RS—such as ROS, reactive nitrogen species (RNS), reactive sulfur species (RSS), methane, ammonia, and carbon monoxide (CO)—are easily diffusible signaling molecules with low molecular weight and high reactive ability [42,43,44,45,46]. These molecules can modify the amino acid residues, particularly cysteine, which alters the protein structure and functions [47]. Moreover, excessive levels of RS are associated with cellular dysfunction due to damage to various cellular components, such as lipids, nucleic acids, and proteins, which can contribute to the progression of chronic diseases [48]. The regulation and complexity of the redox signaling cascade are outlined in a concept known as the reactive species interactome (RSI). The RSI implies the integrated chemical interactions of RS with different components of the downstream signaling cascade and interactions between the RS. The RSI alters cellular, tissue, and organismal levels of flexibility and robustness in response to varying levels of cellular stress and environmental stimuli [43]. Cellular redox homeostasis is largely determined by RS regulation.

G6PD plays a pivotal role in maintaining redox homeostasis. As the rate-limiting enzyme in the PPP, G6PD is responsible for the production of NADPH, a crucial cofactor for various anti-oxidant enzymes. These anti-oxidant enzymes—including superoxide dismutases (SODs), catalase (CAT), glutathione peroxidase 4 (GPX4), thioredoxin (TRX), and reduced glutathione (GSH)—are essential for neutralizing RS such as ROS, RNS, and RSS [49]. NADPH, produced through the PPP, is integral to the function of these anti-oxidant enzymes [49,50]. SODs, with NADPH as a cofactor, catalyze the dismutation of the superoxide anion into hydrogen peroxide. This hydrogen peroxide is then transformed by CAT into water and molecular oxygen, a process that also requires NADPH [51]. GPX4 uses NADPH to convert lipid peroxides into less harmful compounds. Specifically, GPX4 catalyzes the reduction of lipid peroxides by GSH, converting harmful lipid peroxides into their corresponding alcohols and water. This process prevents cell death caused by the accumulation of lipid peroxides, such as ferroptosis [52].

TRX and GSH also depend on NADPH to maintain cellular function by reducing oxidized proteins. TRX acts as a dithiol reductase, reducing protein dithiols through both di-thiol and mono-thiol mechanisms or reducing S-glutathionylated proteins in a GSH-dependent process through its activity as a disulfide oxidoreductase [53]. Together, these processes underscore the pivotal role of G6PD in anti-oxidant activity and in maintaining redox balance. It is noteworthy that G6PD can also exhibit pro-oxidant activity under certain conditions. For instance, in non-tumor cells such as adipocytes, increased G6PD expression has been observed to stimulate oxidative stress and inflammatory responses [54].

Furthermore, recent studies have demonstrated that PPP regulation on redox homeostasis exhibits rhythmic interaction with the circadian clock. The PPP controls circadian oscillations via NADPH metabolism, and inhibition of the PPP alters circadian gene expression, including brain and muscle ARNT-like protein 1 (BMAL1) and circadian locomotor output cycles kaput (CLOCK) [55,56,57]. Given that the circadian clock, an endogenous timekeeper system that controls and optimizes biological processes, plays a vital role in ensuring optimal functioning and health by organizing the behavior and physiology of an organism over the course of the day and night, this interplay between the circadian clock and the PPP—both important for redox homeostasis—presents a complex and dynamic system that is fundamental for understanding diseases.

### 2.3. Role of G6PD in Biomacromolecule Synthesis

Macromolecules, including lipids, nucleotides, and proteins, are crucial components of cellular structure and functions. G6PD plays a significant role in the synthesis of these macromolecules via the PPP. R5P—the end product of the non-oxidative PPP—is a crucial molecule for nucleotide synthesis. Initially, G6P from the glycolysis cascade is converted by G6PD into 6PGL, which is in turn hydrolyzed by the 6-phosphogluconolactonase into 6-phosphogluconate (6PG). This step also releases the first NADPH of the PPP. Another enzyme called 6-phosphogluconate dehydrogenase (6PGD) converts the 6PG into R5P, releasing the second NADPH of the PPP [10].

R5Ps are fundamental molecules for the biosynthesis of purines and pyrimidines, such as adenine, guanine, uracil, thiamine, and cytosine. Initially, R5P induces the production of phosphoribosylpyrophosphate (PRPP) in the purine synthetic pathway. PRPP generates inosine-5′-monophosphate (IMP) through a series of enzymatic reactions. IMP is the primary yield of purine biosynthesis [58] and is converted into adenylosuccinate, which initiates the generation of adenosine monophosphate (AMP) by adenylosuccinate synthase. Another enzyme called adenylosuccinate lyase converts the adenylosuccinate into AMP and releases the fumarate. On the other hand, IMP dehydrogenase catalyzes the IMP and generates xanthosine monophosphate, which is converted into guanine monophosphate (GMP) by GMP synthase with the assistance of glutamine. Subsequently, AMP and GMP are converted into adenosine triphosphate (ATP) and guanosine triphosphate (GTP), respectively, through phosphorylation reactions.

In the initiation of pyrimidine biosynthesis, R5P-derived PRPP is added to a pyrimidine ring. This molecular complex undergoes a series of biochemical reactions to produce orotidine monophosphate (OMP). OMP is then converted into uracil monophosphate (UMP), which in turn is converted into cytosine monophosphate (CMP) and thiamine monophosphate (TMP). Subsequently, UMP, CMP, and TMP are converted into uracil triphosphate (UTP), cytosine triphosphate (CTP), and thiamine triphosphate (TTP), respectively, through phosphorylation reactions [59]. Hence, the G6PD/PPP axis is essential for providing the cells with the building blocks that form DNA and RNA.

In addition to acting as an anti-oxidant, NADPH produced by G6PD/PPP also plays a major role in various stages of lipid biosynthesis by providing the reducing equivalents necessary for the reductive synthesis of lipids from fatty acids. For instance, Wasylenco et al. revealed that each fatty acid requires two and one NADPHs for the elongation and the desaturation phases of the lipid synthesis, respectively [60]. Furthermore, previous studies reported that G6PD and 6PGD, the enzymes responsible for producing the first and second NADPHs of the PPP as described above, are highly expressed in adipose and liver tissues, further confirming the essential role of the PPP in lipid biosynthesis [61].

In the process of lipid production, the NADPH produced through the PPP is used for fatty acid synthesis from acetyl-CoA, which requires two NADPHs per fatty acid. The process begins with the conversion of acetyl-CoA into malonyl-CoA, which in turn is converted into acetoacyl-ACP. After that, one NADPH is required for the conversion of acetoacyl-ACP into β-hydroxyacyl-ACP. Following this, the desaturation phase introduces double bonds into the fatty acid chain to form crotonyl-ACP. Furthermore, in the final stages, NADPH is involved in the reduction of crotonyl-ACP to butyryl-ACP [62]. These reactions are crucial steps in the conversion of acetyl-CoA into fatty acids, which can then be used to synthesize various forms of lipids, including membrane phospholipid, triglyceride, and cholesterol.

In addition to nucleic acid and lipid synthesis, G6PD also regulates protein synthesis. For instance, E4P—another product of the PPP—is involved in the synthesis of aromatic amino acids, including tryptophan, tyrosine, and phenylalanine [63]. Therefore, G6PD plays a crucial role in regulating cellular functions, including proliferation, cell structure, and signal transduction, by providing the building blocks of biomacromolecules.

## 3. Regulation of G6PD Expression

The regulation of G6PD expression is a complex process that occurs at various stages of gene expression, from transcriptional to post-translational levels. This regulation is influential for maintaining the balance of cellular processes, including cell survival and proliferation. Transcriptional regulation of G6PD is achieved by different proteins, such as transcription factors, coactivators, and/or cosuppressors (Table 1).

A number of studies have concluded that the mammalian target of rapamycin 1 (mTOR1) signaling is responsible for the overexpression of G6PD by regulating the binding of Sterol Regulatory Element-binding Protein-1 (SREBP1) with *G6PD* promoter [64,65]. The first intron of *G6PD* accommodates the vitamin D response elements (VDREs). Bao et al. reported overexpression of G6PD in response to the binding of the vitamin D receptor (VDR) to the VDRE [66]. The second intron of *G6PD* contains the binding region of the p53 family protein response element. p53 binds to this region and inhibits the transcriptional activation of *G6PD*. Moreover, p53 can directly bind to the G6PD enzyme to suppress active dimer formation [67]. TAp73, another member of the p53 family with structural homology to p53, can also bind to the second intron and enhances *G6PD* transcription [68,69]. Therefore, while p53 acts as a suppressor of *G6PD* transcription, TAp73 promotes it.

**Table 1:** Proteins regulating G6PD/PPP activity or flux.

Factor	Effect on G6PD	Type of Factor	Type of Regulation	Ref.
YY1	Upregulation	Transcription factor	Transcriptional	[20]
PBX3	Upregulation	Transcription factor	Transcriptional	[21]
SREBP1	Upregulation	Transcription factor	Transcriptional	[64,65]
VDR	Upregulation	Transcription factor	Transcriptional	[66]
c-Myc	Upregulation	Transcription factor	Transcriptional	[70]
HMGA1	Upregulation	Transcription factor	Transcriptional	[71,72]
p65	Upregulation	Transcription factor	Transcriptional	[73]
HIF-1α	Upregulation	Transcription factor	Transcriptional	[74]
p53	Downregulation	Transcription factor	Transcriptional	[67]
p52-ZER6	Upregulation	Transcription factor	Transcriptional	[75]
Snail	Upregulation	Transcription factor	Transcriptional	[76]
Nrf-2	Upregulation	Transcription factor	Transcriptional	[77]
TAp73	Upregulation	Transcription factor	Transcriptional	[69]
NeuroD1	Upregulation	Transcription factor	Transcriptional	[78]
PI3K	Upregulation	Kinase	Post-translational	[79]
AMPK	Downregulation	Kinase	Post-translational	[80]
c-Src	Upregulation	Kinase	Post-translational	[81]
Cyclin D3	Upregulation	Kinase	Post-translational	[81]
PAK4	Upregulation	Kinase	Post-translational	[82]
AKT	Upregulation	Kinase	Post-translational	[79]
Plk1	Upregulation	Kinase	Post-translational	[83]
ATM	Upregulation	Kinase	Post-translational	[84]
PDIA3P	Upregulation	lncRNA	Transcriptional	[85]
PTEN	Downregulation	Phosphatase	Post-translational	[86]
mTORC1	Upregulation	Signaling protein	Transcriptional	[64]
ID1	Upregulation	Signaling protein	Transcriptional	[70]

Our previous findings revealed that *G6PD* transcriptional activity could be directly regulated by Yin Yang 1 (YY1), a zinc-finger transcription factor that is highly conserved in four C2H2 domains and which is predicted to regulate more than 7% of mammalian genes [20,87,88]. YY1 binds to the *G6PD* promoter and acts as a transcriptional activator, thereby enhancing the PPP and subsequently promoting tumorigenesis. Our previous studies also showed that Pre-B-cell leukemia transcription factor 3 (PBX3) and the p52-ZER6 isoform of zinc-finger 398 (ZNF398, also known as ZER6) could promote tumorigenic potential by upregulating *G6PD* transcriptional activity and the PPP [21,75]. Furthermore, Zhang et al. revealed that knocking down *nuclear factor erythroid 2-related factor 2* (*Nrf2*) suppresses the activation of PPP enzymes, including G6PD, in breast cancer [89]. Another study demonstrated that the ID1/Wnt/β-catenin signaling cascade regulates the attachment of c-Myc with the *G6PD* promoter and increases the transcription of *G6PD*, resulting in enhanced cell proliferation and drug resistance for oxaliplatin in hepatocellular carcinoma (HCC) [70]. Furthermore, Yang et al. found that c-Myc interacts with the protein disulfide isomerase family A member 3 pseudogene 1 (PDIA3P), an lncRNA, to regulate G6PD expression. This interaction enhances the DNA binding of c-Myc, promoting its binding to the *G6PD* promoter and leading to increased cell growth and drug resistance in multiple myeloma [85]. Moreover, a recent study on glioblastoma found that the phosphatidylinositol 3-kinase enhancer A (PIKE-A) mediates the binding of the signal transducer and activator of transcription 3 (STAT3) with the *G6PD* promoter, leading to enhanced G6PD expression [90]. Meanwhile, *G6PD* transcription could also be activated by transcription factor p65 as well as hypoxia inducible factor-1α (HIF-1α) stabilized under hypoxia, through their direct binding with the *G6PD* promoter [73,74].

Epigenetics regulation is also important for regulating G6PD expression, since methylation and acetylation in the lysine residues of histone in the *G6PD* promoter significantly affect its transcriptional activity. For instance, H3K9 methylation on the *G6PD* promoter substantially reduces the G6PD expression level [91], while increased histone acetylation, which promotes the G6PD expression level, could be found in various tumors [92].

Post-transcriptional regulation of G6PD is predominantly achieved by the splicing mechanism [93]. Several studies have showed that the splicing mechanism on its pre-mRNA regulates the activity of G6PD in various cancers. Hong et al. found that T-cell leukemia 1 (Tcl1) could interact with heterogenous nuclear ribonucleoprotein (hnRPK) and enhance G6PD pre-mRNA splicing, thereby increasing G6PD expression and, subsequently, the progression of HCC; however, this regulation could be canceled by the inactivation of Tcl1 through phosphorylation by phosphatase and tensin homolog (PTEN)-induced glycogen synthase kinase-3β (GSK3β) [86].

**Figure 3:**
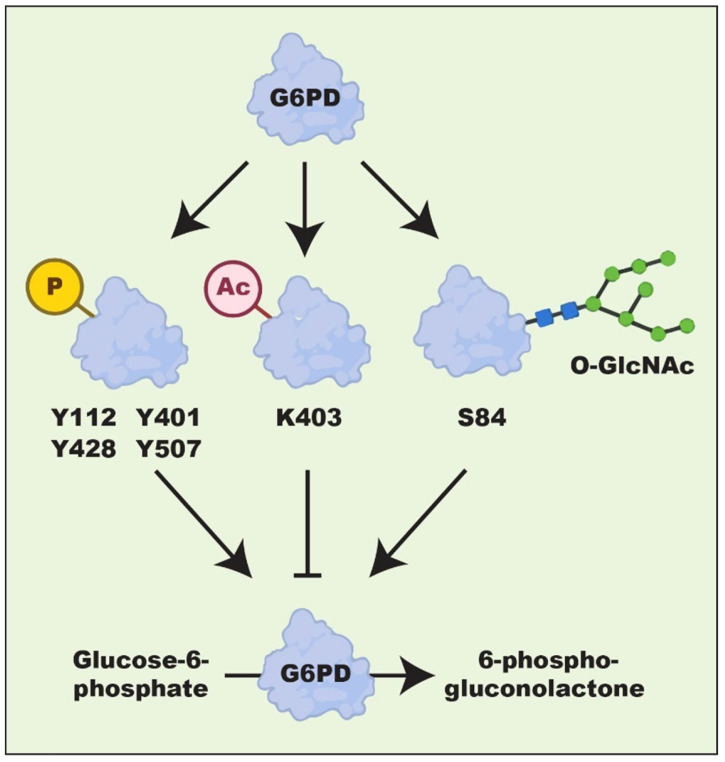
Post-translational modifications of G6PD. Phosphorylation, acetylation, and glycosylation modifications regulate G6PD enzyme activity. P: phosphorylation; Ac: acetylation.

Regulation of G6PD at the post-translational level is primarily orchestrated by epigenetic modifications, including acetylation [94], phosphorylation [83], and ubiquitylation [95]. These post-translational modifications (PTMs) are associated with the activity and stability of the G6PD protein (Figure 3). Acetylation is a crucial regulator of G6PD enzymatic activity. K403 acetylation of G6PD by KAT9/ELP3(acetyltransferase) reduces the activity of the G6PD enzyme [96], whereas NAD-dependent protein deacetylase sirtuin-2 (SIRT2) enhances G6PD function by mediating its deacetylation. This process increases not only the enzymatic activity of G6PD but also its stability, ultimately leading to the progression of HCC [96,97,98,99]. Meanwhile, aspirin—which is known as an antipyretic and analgesic drug—could exert anti-tumor functions by increasing G6PD acetylation-mediated oxidative stress, thereby reducing tumor cell proliferation [100,101].

Phosphorylation modification predominantly occurs on the tyrosine and serine residues [102,103]. G6PD is a substrate of Src family kinases (SFKs), which enhance the G6PD/PPP axis and tumorigenesis by phosphorylating G6PD tyrosine residues, including Y428, Y507, and Y112 [102,103]. Meanwhile, polo-like kinase 1 (Plk1)—a kinase known for its function in cell cycle regulation—can phosphorylate G6PD and promote its dimer formation, resulting in cell cycle activation [83]. Fyn, a member of the SRC family, phosphorylates serine residue at Y401, thereby increasing the enzymatic activity of G6PD [104].

Another important and reversible PTM of G6PD is O-linked β-N-acetylglucosamine (O-GlcNAc), which is known to modify the serine residue. For instance, Rao et al. found that O-GlcNAcylation at Ser84 enhances the binding affinity of NAD^+^ to G6PD [105]. Furthermore, increasing evidence has emerged recently that the enzymatic activity of G6PD is regulated by novel PTMs, including crotonylation, malonylation, propionylation, 2-hydroxyisobutyrylation, glutarylation, β-hydroxybutyrylation, succinylation, and butyrylation [106,107,108,109,110,111]. However, further intensive investigations are needed to reveal the roles of these novel PTMs in the regulation of G6PD and their impacts on cancer progression.

## 4. Role of G6PD in Cancers

The role of G6PD in cancer is primarily delineated through the end- and by-products of the PPP [112]. R5P and NADPH—the two products of the PPP—are critical for supporting accelerated tumor cell proliferation, since they provide the building blocks of nucleotide and lipid biosynthesis [105,113]. Moreover, since NADPH is also pivotal for redox homeostasis, G6PD also plays a significant role in tumor cell survival and drug resistance [83,114]. Indeed, as summarized in Table 2 and described in the next sections, G6PD could enhance tumor growth and survival by maintaining redox homeostasis, suppressing apoptosis induction, increasing drug resistance, and promoting its migration and invasion potential. Increasing evidence suggests that the expression and activity of G6PD have significant influences on different types of cancer (Table 2).

### 4.1. Role of G6PD in Tumor Cell Proliferation

An imbalance of ROS can lead to cell cycle arrest, which can impede cell proliferation. Tumor cells, however, can counteract ROS imbalance through anti-oxidant activity [124]. G6PD/PPP contributes to this process by producing NADPH. G6PD upregulation in tumor cells enhances their anti-oxidant activity, helping in the maintenance of ROS balance, preventing cell cycle arrest, and enhancing proliferation [10,124]. Our previous findings demonstrated that positive regulation of G6PD transcriptional activity by various transcription factors (including YY1, NeuroD1, PBX3, and p52-ZER6) enhances tumor cell proliferation by promoting tumor cell nucleotides and lipid biosynthesis [20,21,75,78].

In addition to G6PD upregulation, G6PD/PPP can also be enhanced through an increase in G6PD enzymatic activity. For instance, phosphorylation, deacetylation, or O-GlcNAcylation modification of the G6PD protein can promote its enzymatic activity and the PPP, resulting in increased cell proliferation [83,96,97,98,99,102,103,104,105]. Furthermore, the activation of the upstream signaling pathways of G6PD can also enhance G6PD/PPP. One study reported that PI3K/AKT activation stabilizes G6PD by inhibiting tripartite motif-containing 21 (TRIM21), an E3 ligase [123]. Moreover, enhanced dimerization of G6PD, either through a knockdown of factors that inhibit G6PD dimerization, such as p53 and *Bcl-2-associated athanogene 3* (*BAG3*) [67,118], or through overexpression of factors that promote G6PD dimerization, such as PLK1 [83], can also enhance G6PD/PPP. Meanwhile, inhibition of G6PD/PPP through *G6PD* knockdown results in decreased tumor cell proliferation [20,21,75,78]. These studies underscore that G6PD, through its role in counteracting ROS imbalance, helps cells avoid cell cycle arrest and enhances proliferation.

### 4.2. Role of G6PD in Tumor Cell Death and Survival

Cell death is a vital biological function for maintaining the number of cells. Resistance to cell death is one of the 14 hallmarks of cancer. Besides the typical types of cell death known previously (i.e., apoptosis and necrosis), several other types of cell death, including ferroptosis and autophagic cell death, have been discovered recently, and their dysregulation is also closely related to tumor development. G6PD plays a significant role in various forms of cell death. For instance, G6PD downregulation has been linked to reduced anti-oxidant activity, which leads to increased cellular oxidative stress and eventually apoptosis [125,126,127,128,129].

Ferroptosis is a form of regulated cell death that is iron-dependent and is characterized by lipid peroxidation [130,131]. Unlike other forms of cell death (such as apoptosis, necrosis, and autophagy), ferroptosis has unique morphological, biochemical, and genetic characteristics, such as mitochondrial shrinkage and accumulation of lipid ROS [130,131]. Several studies have suggested that G6PD serves as a hub gene in the biology of ferroptosis and predicts the poor overall survival of many solid tumors [132,133,134]. Biochemically, G6PD contributes to reductive lipid biosynthesis and the production of GSH, which are crucial for the regulation of ferroptosis [135]. G6PD is also a positive regulator of GPX4, a key gene in ferroptosis regulation. GPX4, in conjunction with GSH, plays a critical role in reducing lipid peroxidation. Specifically, GPX4 acts as a peroxidase that depends on GSH, oxidizing GSH to its oxidative form GSSG, while simultaneously reducing lipid peroxides into their corresponding alcohols [136]. Downregulation of G6PD leads to a decrease in NADPH and GSH levels, which in turn can inhibit GPX4 activity and induce ferroptosis [137].

While known for its significant role in apoptosis and ferroptosis, G6PD may also influence other types of cell death, although this has not been clearly determined. One such type is autophagy, another type of programmed cell death [138]. Autophagy plays a dual role in cellular life and death, acting as both a pro-survival and pro-death process. Pro-survival autophagy promotes tumorigenesis by recycling cellular components through the autophagy-associated lysosomal pathway, while pro-death autophagy (also known as autophagic cell death) leads to cell death [139,140,141]. While the correlation between G6PD and the dual pathways of autophagy has not been totally elucidated, the regulation of autophagy is influenced by glucose metabolism and the redox signaling cascade, suggesting that G6PD could modulate autophagy [142]. Indeed, Mele et al. reported that the inhibition of G6PD can lead to pro-death autophagy, which consequently increases lapatinib-induced cytotoxicity on tumor cells [115]. Thus, increased G6PD expression in tumor cells can enhance their survival through the maintenance of redox homeostasis, which in turn aids in resisting various types of cell death, further underlining the significant role of G6PD in tumorigenesis.

### 4.3. G6PD and Tumor Cell Drug Resistance

Chemotherapy and radiotherapy are the standard treatments for most cancers, despite the existence of other options, such as surgery, hormonal therapy, and gene therapy. These methods are mainly aimed at inhibiting or reducing the abnormal proliferation of tumor cells, many of them by inducing ROS that trigger DNA damage and cell death [143]. However, drug resistance is a major obstacle to achieving the full benefits of these therapeutic strategies, and the molecular and cellular mechanisms behind chemotherapy and radiotherapy resistance are not fully understood. Cellular redox signaling cascades are often associated with drug resistance. Production of NADPH from the PPP and the indirect regulation of GSH by G6PD play pivotal roles in maintaining cellular redox homeostasis. Activation of G6PD/PPP leads to increased anti-oxidant activity to counteract ROS induced by anti-tumor therapies, thus promoting tumor cell drug resistance. Indeed, several studies have demonstrated that G6PD inhibition substantially reverses drug resistance. Polimeni et al. reported increased activity of G6PD and GSH in doxorubicin-resistant HT29 cells upon exposure to oxidative stress; while suppressing the expression of G6PD and the GSH level by dehydroepiandrosterone (DHEA) or 6-aminonicotinamide (6-AN) can increase doxorubicin sensitivity in resistant cells [144,145]. Enhanced activation of G6PD/PPP, which is crucial for retaining the increased level of GSH, has been found in tumor cells resistant to various anti-tumor drugs, including tumor cells resistant to cisplatin, paclitaxel, doxorubicin, lapatinib, tamoxifen, 5-fluorouracil (5-FU), and cytarabine, as reported in different studies [115,116,121,146]. Targeting G6PD with 6-AN can enhance the sensitivity of tumor cells to these drugs, suggesting the potential of combining G6PD inhibitors with conventional anti-tumor drugs [24]. Indeed, a study has demonstrated that combinations of 6-AN and chemotherapy agents 5-FU can inhibit metastases and promote apoptosis in breast cancer [147]; while a combination of doxorubicin and 6-AN can promote the sensitivity of doxorubicin-resistant breast tumor cells to doxorubicin, thereby promoting the overall therapeutic effect [148]. These findings demonstrate that inhibiting G6PD activity can reverse chemotherapy resistance and improve drug efficacy.

### 4.4. Role of G6PD in Tumor Cell Invasion and Metastasis

Another important cancer hallmark is the activation of invasion and metastasis. Tumor cells achieve this by enhancing their invasive capability through epithelial–mesenchymal transition (EMT), a process where tumor cells lose their epithelial properties, gain mesenchymal characteristics, and eventually acquire metastatic properties [149,150]. These properties include enhanced invasion into surrounding tissues through a degrading of the extracellular matrix and the ability to colonize distant organs [149,150].

G6PD is also involved in the regulation of EMT and, eventually, the migration and invasion potential of tumor cells. Zhang et al. showed that the G6PD/HIF-1α/Notch1 axis is upregulated through the overexpression of *Nrf2*, a transcriptional activator of G6PD. G6PD, by maintaining redox homeostasis, aids in the stabilization of HIF-1α, protecting HIF-1α from ROS-mediated deregulation in a hypoxic condition. Subsequently, HIF-1α activates the transcription of *Notch1*, an EMT regulator, leading to the activation of EMT. This subsequently enhances the tumor cell migration and invasion potential [89].

Apart from Notch1, EMT can also be activated through other pathways, such as STAT3, which controls the expression of target genes related to invasive functions like matrix metalloproteinase-2 (MMP-2) and MMP-9. Similarly, G6PD aids in maintaining redox homeostasis, protecting against ROS-mediated STAT3 inactivation, and resulting in increased migration and invasion [119]. Therefore, G6PD-mediated activation of the PPP may promote tumor metastasis through its ability to maintain redox homeostasis.

## 5. G6PD as a Potential Target for Anti-Tumor Therapy

Several studies have shown that inhibiting G6PD using small-molecule inhibitors can reduce cancer progression and chemotherapy resistance. Most studies on cancer have concentrated on two small-molecule inhibitors of G6PD, namely 6-AN and DHEA. In rats, 5–10 mg/kg of 6-AN inhibits G6PD by competing with the coenzyme NADP^+^ for G6PD binding. This competition disrupts the enzyme’s ability to catalyze the conversion of G6P, thereby inhibiting the PPP and reducing the production of NADPH [151]. Furthermore, 6-AN also inhibits 6PGD, which might also contribute to its overall effect on PPP inhibition [152,153,154]. According to several studies, 6-AN has had promising results in terms of increasing the sensitivity of various tumor cells [155]. For example, 1 μM of 6-AN was found to suppress G6PD activity in paclitaxel-resistant ovarian tumor cells, resulting in a higher efficacy of paclitaxel [24]. Furthermore, 6-AN enhances the sensitivity of melanoma and leukemic cells to metformin and cytarabine treatments, respectively, leading to increased apoptosis. Moreover, in bladder cancer, 6-AN-mediated G6PD inhibition suppresses cell proliferation. These findings highlight the potential of 6-AN in anti-tumor therapy. While some side effects, such as vitamin B deficiency and nerve injury, have been reported, the overall therapeutic benefits of 6-AN are promising and warrant further investigation [120,156]. Meanwhile, DHEA—a steroid product from the adrenal gland involved in estrogen and androgen production [22]—inhibits G6PD through uncompetitive inhibition by binding at a site different from the active site, changing the enzyme’s shape, and preventing it from functioning correctly. In doxorubicin-resistant triple-negative breast cancer (TNBC) cells, DHEA treatment can increase doxorubicin cytotoxicity, suggesting a reversal of the chemoresistance phenotype [145].

Besides 6-AN and DHEA, several other novel chemical compounds that can significantly inhibit G6PD have been identified recently, each with unique mechanisms of action. For instance, 200 μM of zoledronic acid, a medication used to treat various bone diseases, inhibits G6PD activity in bladder tumor cells by interfering with the Ras signaling pathway, which is known to regulate G6PD, leading to decreased G6PD expression [157]. Aspirin has demonstrated the ability to reduce NADPH levels and nucleotide synthesis in colorectal tumor cells through acetylation of the G6PD protein, which inhibits its enzymatic activity [101,158]. These findings highlight the diverse range of compounds that can inhibit G6PD, offering multiple avenues for potential therapeutic interventions (Table 3).

It is noteworthy that while G6PD is an attractive target for anti-tumor therapy, merely blocking G6PD might be insufficient [159,160]. Increased activity of G6PD/PPP, which is frequently observed in various tumors, enhances the production of NADPH, which might decrease tumor cell drug sensitivity towards DNA-damage-based anti-tumor drugs such as oxaliplatin, doxorubicin, and daunorubicin [70,144,161,162]. Indeed, previous studies have reported that the combination of G6PD inhibitors with anti-tumor drugs, such as doxorubicin, 5-FU, and paclitaxel, can be a promising strategy in treating tumor and reversing chemotherapeutic resistance [24,144,145,147,148].

Metabolism is a complex process involving more than thousands of enzymes which catalyze a series of precisely controlled reactions. Hence, a single-target approach might not be sufficient to effectively disrupt the metabolic balance in tumor cells. Thus, a multi-target metabolic approach is a possible new option in chemotherapy with higher efficacy. This is in line with the emerging concept that the inhibition of one single enzyme may not be sufficient to block a complex system like cancer. Indeed, emerging evidence showed a better effect of a multi-target metabolic approach for anti-tumor therapy. A multi-target approach, involving simultaneous targeting of multiple enzymes within the same or different metabolic pathways, could be more effective [163,164]. Plant extracts, such as the polyphenol resveratrol, offer a promising option due to their ability to act on multiple cellular targets and influence various metabolic pathways [165,166]. Nevertheless, the potential of G6PD as a target for anti-tumor therapy should not be underestimated, especially when used in combination with other anti-tumor drugs.

## 6. Conclusions

Metabolism is essential for all organisms, since it converts nutrients into energy and building blocks, protects cells from stress, and supports essential life processes such as growth and response to environmental changes. It has become increasingly evident that the metabolic pathways of tumor cells differ from those of normal cells. These alterations enable tumor cells to produce energy even in their severe microenvironment and sustain the excessive demands of rapid proliferation. The crucial functional role of G6PD in influencing tumor cells mainly involves the production of NADPH and R5P, both of which are essential for maintaining redox homeostasis and for biomacromolecule synthesis. G6PD overexpression in tumor cells aids tumor cell proliferation, survival, and invasion, as well as drug resistance. Considering G6PD as a therapeutic target could be a potential way to reduce tumor progression. Furthermore, the intricate mechanisms that regulate G6PD, as well as the metabolic pathways influenced by G6PD, form a complex network that contributes to the survival and proliferation of tumor cells. Given these facts, targeting G6PD could potentially weaken tumor progression.

Targeting G6PD and other metabolic regulators, such as pyruvate kinase isozymes M1/M2 (PKM1/2), monocarboxylate transporter (MCT), and isocitrate dehydrogenase 1/2 (IDH1/2), has attracted attention as a potential anti-tumor therapeutic strategy [167]. Pre-clinical studies have shown potential in using G6PD inhibitors to induce apoptosis in tumor cells. However, challenges remain in G6PD-based anti-tumor therapy. Although DHEA can induce apoptosis in tumor cells through G6PD inhibition, clinical trials of DHEA have been held back due to the high oral dose required and the difficulty in converting DHEA to active androgen. Moreover, whether these inhibitors can effectively reverse the chemoresistance of current clinical drugs used in anti-tumor treatment requires further clinical research. Despite these challenges, the development of potential drugs targeting G6PD with better clinical efficacy remains a promising avenue for anti-tumor therapy. Further research is needed to fully elucidate the mechanism associated with drug resistance and the post-translational modification of G6PD. Furthermore, it remains to be determined whether G6PD might also influence tumor development in ways that are not directly related to its known metabolic and enzymatic functions [168,169]. In conclusion, our review highlights the important role of G6PD in tumorigenesis and as a therapeutic target due to its crucial role in both providing biomacromolecules and maintaining redox homeostasis.

## Figures and Tables

**Table 2:** Expression level and effect of G6PD in different types of cancer.

Tumor	Cell Lines	Phenotypes	Mechanisms	Ref.
Breast	MCF7	Increased lapatinib resistance	Reduced ER-stress-mediated autophagy	[115]
	SUM149, SUM159, T47D, and MCF7	Enhanced proliferation	Histone deacetylase inhibition	[113]
	MCF7, ZR-75-1, T47D, and 293T	Increased tamoxifen resistance	G6PD promoter demethylation	[116]
	MCF7 and MDA-MB-231	Enhanced proliferation, migration, and invasion	Notch-1-mediated EMT activation	[89]
Lung	A549	Reduced cell death	Suppression of ROS-induced apoptosis	[117]
	293T, A549, MCF7, H661, SKOV-3, A375, and U2OS	Enhanced proliferation	G6PD protein O-GlcNAcylation	[105]
Colorectal	HCT116 and HT-29	Enhanced proliferation	G6PD protein acetylation	[101]
	HCT116	Enhanced proliferation	YY1-induced *G6PD* transcriptional activation	[20]
	HCT116	Enhanced proliferation	p52-ZER6-induced *G6PD* transcriptional activation	[75]
	HCT116	Enhanced proliferation	PBX3-induced *G6PD* transcriptional activation	[21]
	HCT116	Enhanced proliferation	NeuroD1-induced *G6PD* transcriptional activation	[78]
	HCT116	Enhanced proliferation	Enhanced G6PD dimerization	[67]
HCC	HepG2	Enhanced proliferation	Enhanced G6PD dimerization	[118]
	HepG2, Huh7, MHCC-97H, HCC-LM3, and L02	Enhanced migration and invasion	STAT3-mediated EMT activation	[119]
Bladder	5637, T24, TCCSUP, and SV-HUC-1	Enhanced proliferation	Suppression of AKT pathway	[120]
Leukemia	MOLM-14	Increased cytarabine resistance	Upregulation of mTORC1	[121]
	HL-60	Enhanced proliferation	SIRT2-mediated G6PD deacetylation	[97]
Glioma	U87-MG and U373-MG	Enhanced proliferation and reduced cell death	SIRT2-mediated G6PD deacetylation	[122]
Cervical	HeLa	Enhanced cell proliferation	Enhanced G6PD dimerization	[83]
Prostate	PC3	Enhanced cell proliferation	Enhanced G6PD protein stabilization	[123]

**Table 3:** Overview of G6PD inhibitors in anti-tumor therapy against different models.

Cancer	Model	Inhibitor	Drug Concentration	Ref.
Breast	MCF7	Polydatin	30 μM	[115]
	MDA-MB-231	DHEA	200 μM	[145]
Colorectal	HCT116 and HT-29	Aspirin	0.25–2.5 mM	[101]
Bladder	5637, T24, TCCSUP, SV-HUC-1	6-AN	10 μM	[120]
	T24, 293T	Zoledronic acid	200 μM	[157]
Leukemia	Mouse	6-AN	5 mg/kg	[121]
Prostate	Mouse	6-AN	1 mg/kg	[123]
HCC	Rat	6-AN	5–10 mg/kg	[151]
